# Accuracy of verbal autopsy, clinical data and minimally invasive autopsy in the evaluation of malaria-specific mortality: an observational study

**DOI:** 10.1136/bmjgh-2021-005218

**Published:** 2021-06-03

**Authors:** Natalia Rakislova, Dercio Jordao, Mamudo R Ismail, Alfredo Mayor, Pau Cisteró, Lorena Marimon, Melania Ferrando, Juan Carlos Hurtado, Lucilia Lovane, Carla Carrilho, Cesaltina Lorenzoni, Fabiola Fernandes, Tacilta Nhampossa, Anelsio Cossa, Inacio Mandomando, Mireia Navarro, Isaac Casas, Khatia Munguambe, Maria Maixenchs, Llorenç Quintó, Eusebio Macete, Mikel Martinez, Robert W Snow, Quique Bassat, Clara Menéndez, Jaume Ordi

**Affiliations:** 1 ISGLOBAL, Hospital Clinic, University of Barcelona, Barcelona, Spain; 2 Department of Pathology, Hospital Clínic, University of Barcelona, Barcelona, Spain; 3 Department of Pathology, Quelimane Central Hospital, Quelimane, Mozambique; 4 Department of Pathology, Maputo Central Hospital, Maputo, Mozambique; 5 Faculty of Medicine, University Eduardo Mondlane, Maputo, Mozambique; 6 Department of Microbiology, Hospital Clínic, University of Barcelona, Barcelona, Spain; 7 Centro de Investigação em Saúde de Manhiça, Manhica, Mozambique; 8 Department of Pediatrics, Maputo Central Hospital, Maputo, Mozambique; 9 Population and Health Unit, KEMRI - Wellcome Trust Research Programme, Nairobi, Kenya; 10 Centre for Tropical Medicine and Global Health, Nuttfield Department of Clinical Medicine, University of Oxford, Oxford, UK; 11 Consorcio de Investigación Biomédica en Red de Epidemiología y Salud Pública (CIBERESP), Madrid, Spain

**Keywords:** malaria, infections, diseases, disorders, injuries

## Abstract

**Background:**

Global malaria mortality estimates are hindered by the low reliability of the verbal autopsy (VA) and the clinical records, the most common sources of information used to estimate malaria-specific mortality. We aimed to determine the accuracy of these tools, as well as of the minimally invasive autopsy (MIA), a needle-based postmortem sampling method, to identify malaria-specific mortality in a large series of deceased patients from Mozambique, using complete autopsy as the gold standard.

**Methods:**

Observational study that included 264 deaths, occurring at a tertiary level hospital in Mozambique, from 1 November 2013 to 31 March 2015 (17 months-long period). Clinical data were abstracted, a computer coded VA was completed using the clinical data as source of information, and an MIA followed by a complete autopsy were performed. Screening for malaria infection was conducted postmortem to all participants using molecular and histological techniques (PCR and immunohistochemistry).

**Findings:**

Malaria infection was considered the cause of death in 6/264 (2.3%) cases: 2/54 children (3.7%, both less than 5 years old) and 4/57 (7.0%) maternal deaths. The sensitivity and specificity of the VA, the clinical data and the MIA to identify malaria-specific deaths were 33.3% and 96.1%, 66.7% and 96.1%, and 100% and 100%, respectively. In addition, malaria was identified as a possible contributor in 14 additional patients who died of other diseases. These cases were also accurately identified by the MIA (sensitivity 82.4%, specificity 100%).

**Interpretation:**

The high sensitivity and specificity of the MIA in identifying malaria may help to improve current estimates of malaria-specific mortality in endemic areas.

**Summary box**

**What is already known?**

- Global malaria mortality estimates rely on the verbal autopsy (VA) and the clinical records, both of these methods being prone to misclassification errors.

**What are the new findings?**

- Malaria was the cause of death (CoD) in 2.3% of fatalities in Maputo (Mozambique).
- Using the complete autopsy as the gold standard for CoD attribution, we found that the sensitivity and specificity of the VA, the clinical data and the minimally invasive autopsy (MIA) to identify malaria-specific deaths were 33.3% and 96.1%, 66.7% and 96.1%, and 100% and 100%, respectively.

**What do the new findings imply?**

- MIA might be a valuable tool to improve estimations of malaria-related deaths in endemic regions.

## INTRODUCTION

Cause-specific mortality information is a cornerstone for health policy-making worldwide.^1^ Unfortunately, civil registration systems and vital statistics are poorly functioning in most low-income and middle-income countries, especially in sub-Saharan Africa, where most countries report only fragmentary and sporadic data and where malaria burden is concentrated.^2 3^ After the launch of the sustainable development goals and the WHO Global Technical Strategy for malaria 2016–2030,^4 5^ malaria-endemic countries have set as mid-term objective to move towards low transmission or pre-elimination status. In this scenario of potentially declining malaria burden, accurate mortality data are crucial to monitor interventions, evaluate progress and adapt strategies. However, obtaining reliable data on malaria-specific mortality, at both national and regional levels, is a major challenge. Indeed, the poor quality and thus reliability of this information seriously hinders the achievement of the Global Technical Strategy goals for malaria 2016–2030.

Most of the available information on malaria-specific mortality is based on data obtained from clinical records or verbal autopsy (VA). However, both methods have consistently shown limitations and are prone to frequent misclassification errors.^6–12^ Indeed, when clinical diagnoses are contrasted with postmortem findings, clinicopathological discrepancies are frequently reported, particularly in settings with limited availability of diagnostic techniques,^13^ and for infectious diseases.^6–9^ Studies conducted in Malawian children dying with a clinical diagnosis of severe malaria showed significant misdiagnosis in up to a quarter of the cases, where an alternative cause of death (CoD) was identified at autopsy.^14^

The VA tool was developed as an indirect approach to establish the CoD by interviewing relatives and witnesses of deaths when other registration methods are unavailable.^15^ However, existing VA methods have also serious limitations in attributing deaths to malaria.^10^ An example of the weaknesses of VA was the marked differences around the global malaria mortality estimates produced by the WHO and the Institute of Health Metrics and Evaluation (IHME),^11 12^ with IHME reporting two times as many deaths in the world due to malaria than WHO estimates. More importantly, up to half of the excess malaria deaths were suggested to occur among adults in Africa,^12^ a finding difficult to credit for most malaria experts. Indeed, it is widely accepted that most malaria-related deaths in stable endemic areas are concentrated in children younger than 5 years,^10 16^ but the contribution of malaria to mortality in older age groups is still unclear.^11 17^

In the last few years, our group has developed a minimally invasive autopsy (MIA) approach, designed to determine the CoD mainly in low-resource settings, as a feasible alternative to the complete diagnostic autopsy (CDA).^18 19^ The procedure leaves hardly any visible trace on the body, and thus, is more acceptable than the CDA.^20^ Furthermore, the MIA can be performed by trained technicians, and can be conducted close to the place where death occurs.^21^ Therefore, this approach could be implemented for mortality surveillance, or as a method to complement information gathered through the VA.^22^ The MIA procedure has been validated in perinatal, paediatric, maternal and other adult deaths in Mozambique and Brazil,^23–27^ showing a moderate to substantial concordance with the CDA, particularly for infectious diseases even when evaluated blindly to any additional data,^23–26^ with such concordance increasing to almost perfect when the clinical information is added.^28^

In the present study, we compare the accuracy of a computerised VA, the clinical records, and the MIA to identify malaria-specific deaths in a large series of in-hospital deaths from Mozambique, comparing the results of these methods against the CDA, the gold standard methodology for CoD attribution.

## Methods

### Study area

The study was conducted at the Maputo Central Hospital (Maputo, Mozambique), a government-funded tertiary-level hospital, which serves as the referral centre for other hospitals in Southern Mozambique. Maputo urban area has low malaria transmission, although some of the peripheral suburbs and surrounding peri-urban and rural areas have moderately stable transmission. Malaria incidence is higher during the rainy season (October to May), and lower during the dry season (June to September).^29^

### Patients included in the study

During the study period (November 2013 to March 2015) 10.296 deaths occurred at the Maputo Central Hospital. All patients included in this study fulfilled the following criteria: (1) a complete autopsy requested by the in-charge clinician and (2) informed consent to perform the autopsy given by the relatives. In order to avoid selection bias, the first two bodies among all deaths eligible for complete autopsy, closest in time of occurrence to 08:00 am were recruited each morning for the study. Thus, 264 of these deaths (2.5% of the total deaths) were included in the study to validate the MIA against the CDA. It comprised 41 neonates, 54 children 1 month to 15 years (31 children under 5 years), 57 maternal deaths (fulfilling the standard definition of the WHO, ie, death during pregnancy or within 42 days of termination of pregnancy irrespective of its cause)^30^ and 112 other adults, including 57 men and 55 non-pregnant women. In all cases an MIA was performed, followed by a CDA on the same body. The general characteristics of each group of patients have been reported elsewhere.^23–26^

### Patient and public involvement

No patients or the public were involved in the study design, recruitment, setting the research questions, interpretation or writing up of results, or reporting of the research.

### Review of the clinical charts and clinical diagnosis

The clinical data from all patients were reviewed and abstracted using a standardised questionnaire. A detailed revision of the entire medical record was conducted with abstraction of demographic data, medical history, admission and hospitalisation files, including signs and symptoms, physical examination, laboratory results, imaging reports and treatments received. All clinical diagnoses registered in the medical record by the clinicians in charge prior to death were included in the list of clinical diagnoses. Although it was assumed that the first diagnosis listed in the clinical record was the principal diagnosis, we considered malaria as a relevant clinical diagnosis, independently of the order in which it was registered.

A specific search for the malaria diagnostic methods conducted during admission (blood smear and/or rapid diagnostic tests) was performed as part of the review of the clinical charts.

### VA diagnosis

We used the InterVA V.4.04 probabilistic model, one of the most commonly implemented VA tools,^31^ which has a good level of agreement with the physician-coded VA, and its interpretation is reproducible and standardised.^32 33^ The InterVA calculates the probability of a set of causes of death given the presence of indicators reported in VA interviews.^34 35^ In this analysis, the information feeding the model was obtained from the clinical record of the patient and from the obstetric record in perinatal deaths, unified into the WHO 2012 VA standard format,^36^ converted into the 245 input indicators of the VA model. We set malaria prevalence to ‘low’, and HIV prevalence to ‘high’ using the InterVA4 package V.1.7.5 implemented in R V.3.5.0 software.^37^

### MIA procedure, analysis and diagnosis

Detailed pathological and microbiological methods of the MIA have been reported elsewhere.^18 19^ The procedure included disinfection of the surface of the body, followed by the collection of blood and cerebrospinal fluid (CSF) and sampling of key solid organs (liver, lungs, central nervous system (CNS), heart, spleen and kidneys), which were analysed through microbiological and pathological techniques.

The histological evaluation included H&E stain in all samples and histochemical and/or immunohistochemical stains whenever required to reach a diagnosis. Microbiological methods, which have been reported in detail elsewhere,^19^ included investigation for highly incident pathogens. This comprised universal detection of antibodies against HIV-1 and HIV-2, multiplexed PCR analyses for most common respiratory viruses and bacteria, bacterial and fungal cultures using samples of blood, CSF, liver, lungs and CNS; and in some cases, further investigation of bacterial or fungal presence using 16S ribosomal RNA (rRNA) gene PCR or the 18S rDNA-ITS PCR, respectively. In patients confirmed to be HIV-positive, an additional microbiological screening was conducted, for common opportunistic pathogens.^23–26^ Other micro-organisms were further investigated depending on the pathological findings observed in the MIA-obtained tissues.

The MIA diagnosis integrated all MIA findings (histology and microbiology), as well as the clinical information.

### CDA procedure, analysis and gold standard diagnosis

Immediately after the MIA, the CDA was performed by another pathologist not involved in the MIA. Histological and microbiological analyses were conducted in samples from the same viscera collected in the MIA and from any grossly identified lesions. The same analytical methods used for the MIA were used for the histological and microbiological samples of the complete autopsy.

The final diagnosis of the CDA integrated all autopsy findings (macroscopy, histology and microbiology), as well as the clinical information and was considered the gold standard for CoD attribution.

### Diagnosis of malaria in the MIA and the CDA and definitions

In all 264 cases, *Plasmodium falciparum* malaria was proactively screened, and parasite density quantified, using a real-time quantitative PCR (qPCR) assay targeting 18S rRNA. Such assay was applied to peripheral blood obtained during the postmortem procedure, and collected as blood spots into Whatman 903 Specimen Collection Paper.^38 39^ Parasitaemia was quantified by extrapolation of cycle thresholds from a standard curve of *P. falciparum* ring-infected erythrocytes.

We selected all the patients with a diagnosis or suspicion of malaria (either clinical, in the VA or in the postmortem examination), and a subset of 10 controls with no suspicion of malaria. In these cases, an immunohistochemical stain was performed in all available tissues with the polyclonal rabbit anti-*P. falciparum* HPRT1/HPRT antibody (LSBio, Seattle, Washington, USA, 30 min incubation, dilution 1/1000), and revealed with Envision FLEX and HRP Magenta as chromogen (Agilent, Santa Clara, California, USA) using the Dako PT Link equipment (Agilent). In all these cases a thorough search of malaria parasites was conducted under immersion oil (1000×) both in the H&E and *P. falciparum* immunohistochemical stains by two of the investigators (NR and JO). Additionally, a thorough search of haemozoin in macrophages was conducted in all tissues from these patients as evidence of the past malaria.

Malaria was considered the CoD (malaria-specific mortality) based on: (a) presence of cerebral malaria or (b) presence of abundant haemozoin deposition in tissues in the absence of other CoD. Malaria was considered a contributor to death on the basis of: (a) parasitaemia detected by PCR and/or (b) presence of malarial pigment in tissues in the presence of another CoD. In this subset of patients, malaria was considered as a possible contributor to death when haemoglobin levels were below 80 g/L (severe anaemia), due to the possible role of malaria in the chain of events leading to death. Malaria was considered as an associated condition death when the haemoglobin levels were within normal limits or showed only mild to moderate anaemia.

### Statistical analysis

The diagnostic performance of the MIA, the clinical records and of the VA to identify malaria as the CoD against the CDA (gold standard) was evaluated as sensitivity, specificity, positive predictive value and negative predictive value, and the 95% CIs for these values were calculated.

Data were analysed with STATA (V.15).

## Results

### Malaria-specific mortality

Malaria was considered the CoD in 6 out of the 264 autopsies (2.3%). Malaria was attributed as the CoD in 2/54 children (3.7%), both cases being in children under-five (2/31, 6.4% of the deaths in children 1 month to 5 years), and in 4/57 (7.0%) maternal deaths. No death directly attributed to malaria was identified among neonates or other adults.

The demographic, clinical and laboratory characteristics of the six deaths caused by malaria are shown in table 1. Fever and neurological symptoms were documented in four of the six patients. Of the four maternal deaths one was a primigravid woman and three were multigravida (two gravid 2, one gravid 3). Three deaths occurred in the third trimester immediately after delivery (36, 37 and 38 weeks of gestation). All three women delivered a dead fetus, two after vaginal delivery and one after caesarean section. The fourth malaria maternal death led to a first trimester abortion (8 weeks of gestation). In none of the malaria-related maternal deaths there was a history of pre-eclampsia.

**Table 1:** Demographic, clinical and laboratory characteristics of malaria-specific deaths

Case	Age	Sex	Age group	Origin *	Malaria † seasonality	HIV	Fever	Neurological symptoms	Other symptoms	Haematocrit/ haemoglobin	Parasitaemia/ rapid test	Clinical diagnosis
1	2	M	Children u-5	Urban	High	–	NA	NA	Dyspnoea	NA	Positive/NA	Severe malaria, sepsis, anaemia
2	3	M	Children u-5	Urban	High	–	Yes	Confusion, agitation	Vomiting, diarrhoea, pallor	NA	NA/NA	Acute gastroenteritis, anaemia
3	23	F	Maternal death	Urban	High	HIV	40	No	Dyspnoea, headache, leucocytosis, low platelet count	22/80	Positive /positive	Malaria
4	28	F	Maternal death	Urban	High	HIV	39	Lethargic, abnormal behaviour, nuchal rigidity	Dyspnoea, pallor, jaundice, leucocytosis low platelet count, hepatosplenomegaly, hypoglycaemic	20/70	Negative/ positive	Malaria
5	28	F	Maternal death	Rural	Low	HIV	No	Coma	Diarrhoea, vomiting, pallor	NA	NA/NA	Haemorrhage postabortion, acute gastroenteritis
6	20	F	Maternal death	Urban	High	–	No	No	Pallor, leucocytosis, low platelet count	24/70	NA/positive	Malaria

*Urban usually corresponds to Maputo city, whereby malaria incidence is generally low. Rural implies other areas where malaria incidence tends to be higher.

†Seasonality for malaria is high during the rainy season (October to May), and low during the dry season (June to September).

F, female; M, male; NA, not available; u-5, under 5 years of age identified by the complete diagnostic autopsy.

Table 2 shows the histological findings of the six malaria-attributable deaths. In five out of the six cases the diagnosis was based on the histological findings (massive sequestration of parasitised erythrocytes in the CNS, consistent with cerebral malaria). In the sixth patient the diagnosis was reached after clinicopathological correlation: identification of isolated CNS parasites and massive pigment deposition in the spleen and liver plus clinical evidence of severe malaria, in the absence of any alternative CoD.

**Table 2:** Pathological features in the complete diagnostic autopsy (gold standard) and the minimally invasive autopsy of malaria-specific deaths

Case	Complete diagnostic autopsy					Minimally invasive autopsy			
	Blood parasitaemia	CNS	Lung	Other	Diagnosis	CNS	Lung	Other	Diagnosis
1	Negative *	Mild parasitaemia	Congestion, haemorrhage	Abundant haemozoin in spleen and liver	Severe malaria (treated)	–	Congestion, haemorrhage	Abundant haemozoin in spleen and liver	Severe malaria, pulmonary haemorrhage
2	271.000	Massive parasitaemia	Massive parasitaemia, haemozoin	Parasites and haemozoin in spleen, liver, heart, kidney, bowel	Cerebral malaria	Massive parasitaemia	Massive parasitaemia, haemozoin	Parasites and haemozoin in spleen, liver, heart, kidney	Cerebral malaria
3	2.879	Massive parasitaemia, oedema	Mild parasitaemia, haemozoin	Parasites and haemozoin in spleen, liver, heart, kidney	Cerebral malaria	Massive parasitaemia, oedema	Mild parasitaemia, haemozoin	Parasites and haemozoin in spleen, liver, heart, kidney	Cerebral malaria
4	34.958	Moderate parasitaemia, oedema	Abundant haemozoin, oedema	Parasites in spleen. Abundant haemozoin in spleen, liver	Cerebral malaria	Moderate parasitaemia, oedema	Abundant haemozoin, oedema	Parasites in spleen. Abundant haemozoin in spleen, liver	Cerebral malaria
5	212.308	Moderate parasitaemia	Massive parasitaemia, haemozoin, oedema	Parasites in spleen, liver, heart. Abundant haemozoin in spleen, liver	Cerebral malaria	Moderate parasitaemia	Massive parasitaemia, haemozoin, oedema	Parasites in spleen, liver, heart. Abundant haemozoin in spleen, liver	Cerebral malaria
6	176.738	Massive parasitaemia	Massive parasitaemia, haemozoin	Parasites in spleen, liver, kidney, uterus. Abundant haemozoin in spleen, liver	Cerebral malaria	Massive parasitaemia	Massive parasitaemia, haemozoin	Parasites in spleen, liver, kidney	Cerebral malaria

*Patient had received antimalarial treatment prior to arrival.

CNS, central nervous system.

**Figure 1:**
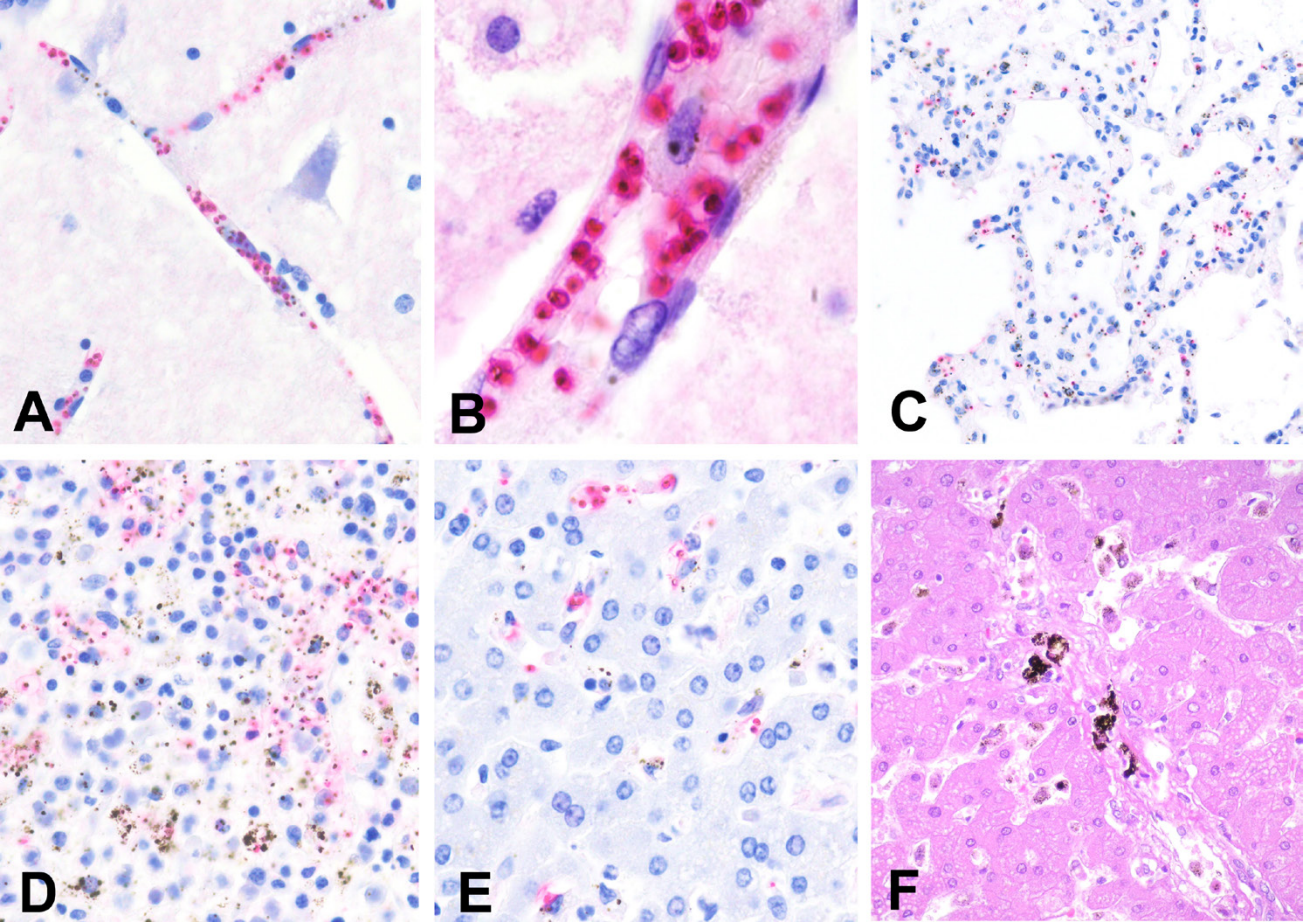
Massive sequestration of *Plasmodium falciparum* infected erythrocytes in the capillaries of the central nervous system. (A) Cortical vessel showing many parasitised erythrocytes (200×). (B) High power field of single capillaries showing massive parasitised erythrocytes adherent to the endothelium (1000×). Clear haemozoin pigment dots are clearly seen in almost all parasites, indicating that are mature forms. (C) Lung, showing abundant parasitised erythrocytes in the septal capillaries (200×). (D) Spleen showing abundant parasitised erythrocytes in the sinusoids of the red pulp. (E) Liver showing abundant parasitised erythrocytes in the sinusoids (200×). (A–E) Immunohistochemical stain, polyclonal rabbit anti*-P. falciparum* HPRT1/HPRT antibody. (F) Past malaria with abundant macrophages with malarial pigment in the portal tracts (H&E, 200×).

The immunohistochemical staining revealed, in addition to the parasitised erythrocytes in the CNS capillaries, the presence of the parasitised erythrocytes in the splenic sinusoids in five cases, in the capillaries of the septal alveoli of the lung, in the liver sinusoids in four patients and in the capillaries of the heart and kidney in three cases. Lung oedema was identified in two patients. In one case there was massive sequestration of parasitised erythrocytes in the intestinal capillaries. Most parasitised erythrocytes sequestered in the microvasculature of both the CNS and other viscera were mature stages (trophozoites and schizonts), characterised by the presence of pigment dots in the cytoplasm. Several histological examples of severe malaria cases are shown in figure 1. No other plausible CoD was identified in any of the six deaths caused by malaria.

No parasites were identified in the immunohistochemical study in any of the 10 cases selected as controls.

### Malaria as contributor to death

Three additional cases tested positive for *P. falciparum* by PCR. One case was a congenital malaria identified in a neonate who showed high blood parasitaemia in the qPCR analysis. Neither parasites, nor malarial pigment were identified in the tissues in the histological and immunohistochemical stains, and a neonatal bacterial sepsis was identified as the CoD. In two cases low blood parasitaemia levels were detected by qPCR (two children aged 7 and 10 years, respectively).

Evidence of past malaria (haemozoin in the liver and the spleen) was identified in further 10 cases (six children and four maternal deaths). No other malaria-related findings were observed, and no parasites were identified in any of these cases through immunohistochemistry. In all these cases a pathologically conclusive CoD different from malaria was identified.

Table 3 shows the demographic, clinical and laboratory characteristics, as well as the evidence of malaria at the CDA and the final CoD of these 13 patients, who died of other diseases but in whom malaria had possibly contributed to death. Seven of these cases had severe anaemia on the basis of haemoglobin levels below 80 g/L, and malaria was considered as a significant contributor to death.

**Table 3:** Clinical and pathological features of the cases with malaria as a contributor to death, in patients who died of other diseases

Case	Age	Sex	Age group	Origin*	Malaria † seasonality	HIV	Haematocrit/ Haemoglobin	Parasitaemia/ rapid test	Cause of death	Evidence of malaria at autopsy	Detected by MIA
**Patients with severe anaemia (malaria as a possible contributor to death**)											
8	7	M	Children 6—15	Rural	High	HIV	23/70	Negative/ negative	Burkitt lymphoma	PCR parasitaemia (low)	Yes (PCR parasitaemia, low)
9	10	M	Children 6—15	Urban	High	–	9/30	Negative/ negative	Sepsis due to Gram-negative bacteria	PCR parasitaemia (low)	Yes (PCR parasitaemia, low)
11	10	M	Children 6—15	Rural	High	–	9/20	Negative/ negative	Sepsis due to Gram-negative bacteria	Haemozoin in liver, lung and spleen	No
15	2	M	Children u-5	Urban	High	HIV	21/70	NA/negative	Sepsis (Gram-negative bacteria)	Haemozoin in liver and spleen	Yes (haemozoin in liver and spleen)
16	27	F	Maternal death	Urban	High	HIV	18/60	Negative/NA	Genital tract infection following abortion	Haemozoin in lung	No
17	27	F	Maternal death	Urban	High	HIV	14/50	Negative/NA	Liver failure	Haemozoin in spleen	No
19	27	F	Maternal death	Urban	High	–	18/60	NA/negative	Obstetric haemorrhage	Haemozoin in liver	Yes (haemozoin in liver)
**Patients with mild to moderate anaemia or with no available data (malaria as associated condition**)											
7	0	F	Neonate	Urban	High	–	41/140	NA/NA	Neonatal sepsis, congenital malaria	PCR parasitaemia (107,167)	Yes (PCR parasitaemia, 107,167)
10	4	M	Children u-5	Urban	Low	–	28/90	Negative/ negative	Encephalitis	Haemozoin in liver and spleen	Yes (haemozoin in liver and spleen)
14	13	M	Children 6—15	Urban	High	–	41/130	NA/negative	Sepsis (Streptococcus pneumoniae)	Haemozoin in liver and spleen	Yes (haemozoin in liver and spleen)
12	6	F	Children 6—15	Urban	High	–	33/110	Negative/NA	Encephalitis	Haemozoin in liver and spleen	Yes (haemozoin in liver and spleen)
13	4	M	Children u-5	Rural	Low	–	30/100	NA/NA	Burkitt lymphoma	Haemozoin in liver	Yes (haemozoin in liver)
18	32	F	Maternal death	Urban	High	–	NA	NA/NA	Obstetric haemorrhage	Haemozoin in liver	Yes (haemozoin in liver)

*Urban usually corresponds to Maputo city, whereby malaria incidence is generally low. Rural implies other areas where malaria incidence tends to be higher.

†Seasonality for malaria is high during the rainy season (October to May), and low during the dry season (June to September).

F, female; M, male; MIA, minimally invasive autopsy; NA, not available; u-5, under 5 years of age.

### MIA diagnosis of malaria

The histological findings of the MIA of the six deaths caused by malaria are shown in table 2. The MIA identified all six malaria-specific deaths, and correctly diagnosed all cerebral malaria. Finally, 4/7 cases with malaria as a probable contributor to death and all six cases in which malaria was considered a possible contributor to death were diagnosed by the MIA.

### Clinical and VA diagnosis of malaria

The demographic characteristics, the clinical features, the laboratory data and the final CDA diagnosis of the patients clinically diagnosed of malaria or classified as malaria-specific deaths by the VA are shown in table 4.

**Table 4:** Demographic characteristics, clinical features, laboratory data and final diagnosis (gold standard) of the patients with clinical diagnosis of malaria or diagnosed of malaria by verbal autopsy

Case	Age	Sex	Age group	Origin	Malaria * incidence	Previous conditions	Fever	Haematocrit/ Haemoglobin	Parasitaemia/ rapid test	Cause of death
**Clinical diagnosis of malaria**										
3	23	F	Maternal death	Urban	High	HIV	Yes	22/80	Positive/positive	Cerebral malaria
4	28	F	Maternal death	Urban	High	HIV	Yes	20/70	Negative/positive	Cerebral malaria
6	20	F	Maternal death	Urban	High	–	No	24/70	NA/NA	Cerebral malaria
11	10	M	Children 6–15	Rural	High	–	Yes	9/20	Negative/negative	Gram-negative sepsis‡
12	6	F	Children 6–15	Urban	High	–	Yes	33/110	Negative/NA	Encephalitis‡
20	34	F	Maternal death	Urban	High	–	No	29/100	NA/NA	Streptococcal sepsis
21	17	M	Adult	Urban	High	–	Yes	34/120	Negative/NA	Pneumococcal meningitis
22	40	F	Adult	Urban	High	HIV	Yes	22/80	Negative/NA	Cytomegalovirus pneumonitis
**Clinical diagnosis of malaria and verbal autopsy classified as malaria**										
1	2	M	Children u-5	Urban	High	–	NA	NA/NA	Positive/NA	Severe malaria
23	33	F	Adult	Urban	High	–	Yes	NA/NA	NA/NA	Gastroenteritis
24	25	M	Adult	Urban	High	HIV	Yes	44/160	Negative/positive	Disseminated cryptococcosis
25	50	M	Adult	Urban	High	HIV	Yes	NA/NA	NA/NA	Pneumococcal meningitis
**Verbal autopsy classified as malaria**										
2	3	M	Children u-5	Urban	High	–	Yes	NA/NA	NA/NA	Cerebral malaria
16	27	F	Maternal death	Urban	High	HIV	Yes	18/60	Negative/NA	Puerperal sepsis‡
19	27	F	Maternal death	Urban	High	–	No	18/60	NA/negative	Haemorrhage postabortion‡
26	0	M	Children u-5	Urban	High	–	Yes	18/60	Negative/negative	Pneumococcal meningitis
27	28	F	Maternal death	Urban	High	HIV	No	14/50	Negative/negative	Liver failure
28	16	F	Maternal death	Urban	High	–	Yes	10/30	NA/NA	Liver failure
29	28	F	Adult	Urban	High	–	Yes	12/40	Negative/negative	Bacterial sepsis
30	37	M	Adult	Rural	High	HIV	Yes	NA/NA	NA/NA	Bacterial sepsis

*Urban usually corresponds to Maputo city, whereby malaria incidence is generally low. Rural implies other areas where malaria incidence tends to be higher.

†Seasonality for malaria is high during the rainy season (October to May), and low during the dry season (June to September).

‡Histological evidence of past malaria (haemozoin accumulation in liver and spleen) identified in the autopsy.

F, female; M, male; NA, not available; u-5, under 5 years of age.

Malaria was clinically diagnosed premortem in 12 patients. In nine of them, malaria was the first clinical diagnosis, whereas in three cases malaria was included in the list of additional diagnoses. Of them, eight patients had received antimalarial drugs. In 11/12 patients the temperature was recorded in the clinical records; 9 of these 11 patients (82%) had fever. One case was a child less than 5 years old, two were children aged 6–15 years, four were maternal deaths and five were other adults. The CDA confirmed a death caused by malaria in four cases whereas in the remaining cases an alternative CoD was identified.

A blood smear for malaria investigation had been performed during admission in 115/264 patients (43.6%) and was positive in four cases. A rapid diagnostic test had been performed in 98/264 patients (37.1%) and was positive in six patients. Two patients had a positive result for both tests (tables 1, 3 and 4).

Twelve cases were classified as malaria by the VA. In 10 cases, malaria was the only diagnosis provided, whereas in two cases, malaria infection was the second probable CoD. Three cases classified by the VA as malaria-specific deaths were children under-five years of age, four were maternal deaths and five were other adults. The CDA confirmed a death due to malaria in two cases, whereas in the remaining cases an alternative CoD was identified.

Fever was documented in 97 patients (5/41 neonates, 16/31 children under-five, 10/23 children 6–15, 17/57 maternal deaths and 49/112 adults). Nine per cent of the febrile deaths were considered clinically and by the VA as deaths caused by malaria (0% and 13%, respectively of the febrile deaths occurring in children under-five, 20% and 0% of the children 6–15, 12% each or the maternal deaths and 10% each of the adult deaths). The CDA confirmed malaria-related death in only 3% of the cases (6% of the febrile deaths occurring in children under-five and 12% of the maternal deaths).

### Sensitivity and specificity of the clinical records, the VA and the MIA for the diagnosis of malaria as CoD

Table 5 shows the sensitivity, specificity, positive and negative predictive values of the MIA, the clinical data and the VA in identifying malaria-specific deaths and for all deaths possibly related to malaria, which included the malaria-specific deaths and the deaths in which malaria was a possible contributor (patients with evidence of malaria and severe anaemia). The MIA had a sensitivity and specificity of 100% to identify malaria-specific deaths. Malaria as a possible contributor to death was identified by the MIA with a sensitivity of 82.4%, and a specificity of 100%. These cases of malaria as a possible contributor were not identified clinically nor by the VA, with the exception of two cases that were mistakenly classified as deaths due to malaria by the clinicians and of two by the VA.

**Table 5:** Sensitivity, specificity, positive and negative predictive value of the verbal autopsy, clinical and minimally invasive autopsy diagnosis for malaria-specific deaths and for all deaths probably associated with malaria (malaria specific deaths plus patients with any evidence of malaria infection at autopsy and with severe anaemia)

	Sensitivity	Specificity	Positive predictive value	Negative predictive value
Malaria-specific deaths				
Verbal autopsy	33.3% (4.3 to 77.7)	96.1% (93.0 to 98.1)	16.7% (2.1 to 48.4)	98.4% (96.0 to 99.6)
Clinical diagnosis	66.7% (22.3 to 95.7)	96.1% (93.0 to 98.1)	28.6% (8.4 to 58.1)	99.2% (97.1 to 99.9)
Minimally invasive autopsy	100% (54.1 to 100)	100% (98.6 to 100)	100% (54.1 to 100)	100% (98.6 to 100)
Deaths probably associated with malaria				
Verbal autopsy	30.8% (9.1 to 61.4)	96.8% (93.8 to 98.6)	33.3% (9.9 to 65.1)	96.4% (93.3 to 98.4)
Clinical diagnosis	38.5% (13.9 to 65.4)	96.4% (93.3 to 98.3)	35.7% (12.8 to 64.9)	96.8% (93.8 to 98.6)
Minimally invasive autopsy	76.9% (46.2 to 95.0)	100% (98.5 to 100)	100% (69.2 to 100)	98.8% (96.6 to 99.8)

The figures in parenthesis indicate 95% CIs.

## Discussion

To our knowledge, this is the first time that the MIA method has been compared with the currently used methods to ascertain the CoD (the VA and the clinical records) and shown higher sensitivity and specificity (100% and 100%, respectively) in identifying malaria-specific mortality. All patients with massive sequestration of *P. falciparum* parasites in the cerebral capillaries, the pathological hallmark of cerebral malaria and the most conclusive evidence of malaria-specific death both in adults and children, were clearly identified by the MIA. Interestingly, whereas 9% of all febrile deaths were attributed to malaria by the clinicians and the VA, the CDA confirmed malaria-specific death in only 3% of the patients, with marked discrepancies observed particularly in children other than 5 years and adults, which is in keeping with recent data showing a decrease in the malaria test positivity rate in adults.^40^ Finally, as observed in previous studies,^14 41 42^ the sequestration of parasitised erythrocyte was not restricted to the CNS, but observed in other organs as well.

In this study, two out of the six malaria-specific deaths were clinically missed, yielding a sensitivity of only 66.7% for the clinical data to ascertain this infection as the CoD. Additionally, the clinicians incorrectly diagnosed 10 cases as malaria. In these 10 cases other causes of death were identified in the CDA (and by the MIA), and no evidence of malaria was found (specificity of 96.1%). The challenges of accurately diagnosing infectious diseases in low resource settings have been highlighted in several studies, with increasing frequency of clinical errors when ancillary diagnostic tests are limited,^6–9^ a frequent situation in low-income and middle-income countries, including malaria-endemic countries. This study highlights the urgent need of increasing the diagnostic capacity in these settings, which could, not only improve current data on CoD, but more importantly reduce mortality.

The InterVA model missed four of the six malaria-specific deaths (sensitivity of 33.3%). Remarkably, although the InterVA model correctly identified the two paediatric cases, it missed out all maternal deaths caused by malaria, indicating that this VA model has limitations in correctly identifying malaria-specific deaths in this high-risk group. On the other hand, 10 patients classified by the model as malaria-related deaths had alternative causes of death in the CDA and no postmortem evidence of malaria infection. Interestingly, although the VA was fed using clinical data, only four cases were classified as deaths caused by malaria both clinically and by the VA, whereas eight cases not diagnosed clinically as malaria were classified as malaria-specific deaths by the VA. Overall, VA validation studies have shown low levels of sensitivity (19%–75%) and specificity (69%–100%) for attributing malaria infection as CoD.^43–49^ Reasons for the low diagnostic performance of the VA in attributing malaria as a CoD include its poor specificity for diseases with overlapping clinical symptoms, such as fever and neurological or respiratory symptoms. This is the case of malaria infection, meningitis, pneumonia, sepsis, as well as opportunistic HIV-related infections, all of these infections being highly prevalent in many sub-Saharan African countries.^50 51^ Finally, the VA is markedly influenced by the malaria epidemiology, and in endemic areas it tends to assign to malaria any acute febrile illness in the absence of an obvious alternative cause.^44^

In addition to accurately identifying malaria-specific deaths, the MIA captures with relatively high precision patients who were considered to die of other diseases but who had low-level parasitaemia, or evidence of past malaria infection, and in whom malaria may have contributed to death, including patients with severe anaemia in whom malaria was a probable contributor to death (sensitivity 76.9%, specificity 100%). Most of these cases were either not identified, or wrongly classified as malaria-specific deaths clinically and by the VA. The difficulties of assigning malaria as an underlying, contributing or indirect CoD have been noted,^10^ and the MIA could provide relevant information in this challenging area. In malaria-endemic areas, the progressive acquisition of natural immunity allows the common occurrence of malarial infection without clinical symptoms. Such malaria ‘tolerance’ is a confounding factor when investigating disease and death, as the detected parasitaemia cannot be ruled out as the aetiology of the disease and death. The MIA may offer an opportunity to improve our knowledge on the indirect contribution of malaria to death.

In this study, malaria-specific deaths occurred only in two groups, namely, children younger than 5 years of age and pregnant women. Indeed, although some reports suggest that cerebral malaria is a common cause of maternal mortality in areas of stable transmission,^42 52^ it is generally considered that malaria-specific maternal deaths are largely restricted to low and unstable malaria transmission areas.^17^ In this regard, studies focusing at determining the real contribution of malaria to maternal mortality are warranted in areas with different malaria transmission. Interestingly, none of the malaria-specific deaths occurred in children aged 6–15 or in adults other than maternal deaths, which is in contrast with some reports suggesting that malaria is responsible for up to 25% of deaths in children 6–15 years old and a significant number of deaths in adults,^15 53 54^ particularly among elderly people.^55 56^ Moreover, no case of low-level parasitaemia or past malaria was identified among adults, which may be an indirect evidence of the limited contribution of malaria to mortality in this age group.

Our study has several strengths. First, it evaluates the performance of the VA, the clinical records and the MIA against the CDA, the gold standard for CoD attribution. Notably, the CDA included an extensive microbiological testing comprising specific qPCR for malaria infection. Another strength is that all age groups were included in the analysis. The main limitations of the study include a relatively small number of cases, particularly of some age groups, like young children, which may partly explain the small number of malaria-specific deaths. However, the complexity of the CDA and the scarcity of specialised personnel preclude a widespread use of these studies. In addition, the small number of malaria-specific deaths identified in our study may be considered as a limitation. Further studies in high malaria burden settings are warranted to confirm our results. Also, the fact that the study is based in a tertiary hospital might be seen as a limitation to extrapolate findings to deaths occurring in rural health facilities or in the community, since causes of death in rural settings may be different to that of those occurring in a hospital. However, the main objective of this study was evaluating the MIA against a reliable gold standard. Finally, the VA results might have been influenced by the fact that the indicators used to estimate the CoD by the VA model were extracted from medical records, which may have resulted in some missing indicators. Nevertheless, most of these missing indicators were secondary questions related to the duration of the event.^57^

In conclusion, this study provides evidence on the high sensitivity and specificity of the MIA method in identifying malaria-specific deaths. It also shows that the VA and the clinical records have serious limitations in recognising malaria infection as CoD in an endemic setting. The MIA, implemented in selected surveillance sites, could be used to help clinicians to reduce diagnostic errors, to improve the quality and performance of current VA tools, to mathematically modulate the data provided by the VA, and ultimately, to improve all-cause and disease-specific statistics.

## Data Availability

All data relevant to the study are included in the article.
